# Host Plants Affect the Foraging Success of Two Parasitoids that Attack Light Brown Apple Moth *Epiphyas postvittana* (Walker) (Lepidoptera: Tortricidae)

**DOI:** 10.1371/journal.pone.0124773

**Published:** 2015-04-20

**Authors:** Yi Feng, Steve Wratten, Harpinder Sandhu, Michael Keller

**Affiliations:** 1 School of Agriculture, Food and Wine, University of Adelaide, Adelaide, Australia; 2 Bio-Protection Research Centre, Lincoln University, Lincoln, New Zealand; 3 School of the Environment, Flinders University, Adelaide, Australia; University of California, Berkeley, UNITED STATES

## Abstract

The light brown apple moth, *Epiphyas postvittana* is a key pest of wine grapes in Australia. Two parasitoids, *Dolichogenidea tasmanica* and *Therophilus unimaculatus*, attack the larval stage of this pest. *D*. *tasmanica* is dominant in vineyards, whereas *T*. *unimaculatus* is mainly active in native vegetation. We sought to understand why they differ in their use of habitats. Plants are a major component of habitats of parasitoids, and herbivore-infested plants influence parasitoid foraging efficiency by their architecture and emission of volatile chemicals. We investigated how different plant species infested by *E*. *postvittana* could affect the foraging success of the two parasitoid species in both laboratory and field experiments. Four common host-plant species were selected for this study. In paired-choice experiments to determine the innate foraging preferences for plants, both parasitoid species showed differences in innate search preferences among plant species. The plant preference of *D*. *tasmanica* was altered by oviposition experience with hosts that were feeding on other plant species. In a behavioral assay, the two parasitoid species allocated their times engaged in various types of behavior differently when foraging on different plant species. For both parasitoids, parasitism on *Hardenbergia violacea* was the highest of the four plant species. Significantly more larvae dropped from *Myoporum insulare* when attacked than from the other three host-plant species, which indicates that parasitism is also affected by interactions between plants and host insects. In vineyards, parasitism by *D*. *tasmanica* was significantly lower on *M*. *insulare* than on the other three host-plant species, but the parasitism rates were similar among the other three plant species. Our results indicate that plants play a role in the habitat preferences of these two parasitoid species by influencing their foraging behavior, and are likely to contribute to their distributions among habitats.

## Introduction

Successful parasitism by parasitoids begins with a series of host-searching behaviors that lead females to locate their potential hosts. This process includes habitat location, host location, and host acceptance [1]. Parasitoid searching behavior is under strong natural selection pressure, because successful foraging is directly linked to reproduction [2,3]. Host-searching behavior determines the efficiency of parasitoids, and thus understanding it is a key element in evaluating their suitability as biological control agents [4].

Herbivore-infested plants influence the foraging efficiency of parasitoids in various ways [5,6]. For example, herbivore-induced volatiles emitted by the host plant are key signals that lead parasitoids to their hosts [7,8]. Herbivore-infested host plants can selectively attract natural enemies [9] and, in some cases when different plant species are infested with the same herbivore, some species may attract more parasitoids than the others, resulting in higher parasitism rates under natural conditions [10]. In addition to plant volatiles, plant architecture can also influence the interactions between the parasitoids and their hosts [11–16]. Plant morphological characteristics such as plant-surface structural complexity [17], presence of dense trichomes [18,19], and leaf surface area [14,20,21] can significantly influence the success rates of parasitoids or predators in finding their hosts. To understand how plants affect parasitoid foraging efficiency, the effects of plant attributes including both plant volatiles and other characteristics like architecture should be considered.

Parasitoids may also rely on both innate mechanisms and learned cues associated with host availability during foraging [22]. Through learning, parasitoids can adaptively optimize their foraging efficiency by altering their innate preferences [23]. The effects of learning on the ability of parasitoids to locate hosts have been documented in both laboratory [24,25] and field studies [26]. For instance, foraging experience on different herbivore-infested cabbage varieties can lead generalist parasitoids to have a preference for the herbivore-infested plant species that they have experienced [24]. However, when indigenous parasitoids forage for hosts on a wide range of plant species involving both native and exotic plants, it is not known whether previous oviposition experience on one plant species will influence their subsequent foraging preference in favor of the same species.

In agro-ecosystems, native plant species happen to grow within or adjacent to the crop plants, which are mostly non-native introduced species. To investigate the effect of plant species on the foraging efficiency of generalist parasitoids, both the plant attributes and the adaptive learning behavior of the parasitoids should be considered.

**Fig 1:**
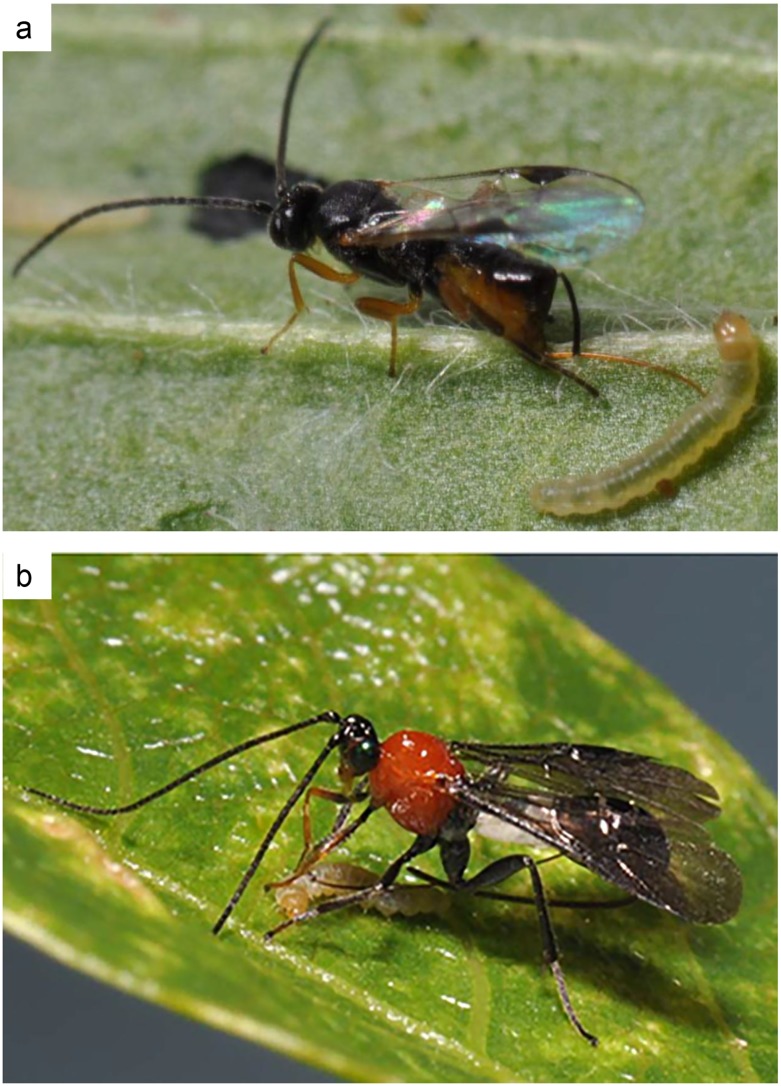
Braconid parasitoids, *Dolichogenidea tasmanica* (a) and *Therophilus unimaculatus* (b), stinging second-instar *Epiphyas postvittana*. These actively foraging parasitoids are generalists that attack a range of leafrollers (Lepidoptera: Tortricidae). Photos by Michael Keller (a), and Yi Feng (b).

In this study, we investigated the foraging behavior of *Dolichogenidea tasmanica* (Cameron) (Hymenoptera: Braconidae) and *Therophilus unimaculatus* (Turner) (Hymenoptera: Braconidae) (Fig 1). Both species are indigenous to Australia and are solitary, koinobiont, generalist endoparasitoids [27–29]. They both attack the light brown apple moth, *Epiphyas postvittana* (Walker) (Lepidoptera: Tortricidae), which is a native Australian, leaf rolling, polyphagous, multivoltine moth. *Epiphyas postvittana* is the key insect pest of grapevines in Australia [30], and it also attacks plants from 123 genera in 55 plant families in this country. Among these plant species, 22 are from native genera, while 101 are from exotic genera [31]. Approximately 25 hymenopteran parasitoids are reported to be associated with *E*. *postvittana* in Australia [32], however, *D*. *tasmanica* and *T*. *unimaculatus* are the predominant parasitoids of *E*. *postvittana*. The former is the most abundant larval parasitoid in vineyards, while the latter one is most common in the adjacent vegetation [33]. It is not known why one parasitoid species is more active in vineyards while the other one is not. Plants could play a key role among a number of factors that affect the activity of these parasitoids in vineyard ecosystems. However, it is not known how different plant species in and around the vineyards affect their foraging behavior and habitat associations of these two parasitoids.

We investigated (1) whether female *D*. *tasmanica* and *T*. *unimaculatus* have innate search preference for different host plant species infested with larval *E*. *postvittana*; (2) whether previous oviposition experience alters the host-plant preferences of the parasitoids; (3) how different plant species infested with *E*. *postvittana* affect the behavior and foraging efficiency of the two parasitoids; and (4) whether parasitism is affected by different plant species in vineyards. We first tested the in-flight preference of both parasitoid species for host-infested plants with dual-choice wind tunnel assays. Four representative plants species, which included both native and exotic species, were selected. We then tested whether previous oviposition experience on one plant species could influence the in-flight preference of *D*. *tasmanica* for host plants. Next, we investigated the searching behavior of two parasitoids on four plant species. Finally, a field experiment was conducted to determine whether parasitism of larval *E*. *postvittana* was influenced by their host plants in vineyards. Overall, we gained insights into how plant species affect the foraging success of two generalist parasitoids that attack *E*. *postvittana*.

## Materials and Methods

Ethics Statement: Permission to conduct the field experiment was granted by the vineyard managers Geoff Hardy and David Hamilton. No permit was required for the laboratory studies.

### Insects and Plants

An artificial diet was used to rear *E*. *postvittana* [34] at 22 ± 2°C under a 12 L:12 D light: dark cycle in an insect-rearing room. This culture has been maintained for 200 generations, with the annual addition of field-collected individuals to maintain genetic diversity. The colonies of *D*. *tasmanica* and *T*. *unimaculatus* were originally established from parasitized leafrollers collected in a vineyard (35°16'05'' S, 138°37'10'' E) near Adelaide, Australia in November 2011. These parasitoids were reared on larval *E*. *postvittana* that fed on plantain (*Plantago lanceolata* L.) and were maintained in cages at 23 ± 2°C with a relative humidity of 60 ± 10% under a 14 L: 10 D light: dark cycle. Naturally occurring larval *E*. *postvittana* were collected from the field (35°58'18'' S, 138°38'32'' E) every two months and the newly emerged adult parasitoids were added to the respective colonies.

We chose four plant species that have been reported to be common host-plants for leafrollers in Australia [31] representing three categories: (1) an introduced economic crop, wine grape, *Vitis vinifera* L., cv. Chardonnay, which is highly susceptible to attack by *E*. *postvittana* [35]; (2) two Australian native perennial plants, *Hardenbergia violacea* (Schneev.) Stearn and *Myoporum insulare* R. Br.; and (3) an exotic ground cover species, plantain, *Plantago lanceolata* L. These plant species differ in their architecture and the level of protection available to larval *E*. *postvittana*.

Experimental plants were grown in containers. *P*. *lanceolata* was grown from seed three months prior to the experiment. The Chardonnay grape vines were grown from pencil-sized cuttings collected from a vineyard during winter. Native plants (*H*. *violacea* and *M*. *insulare*) (~20 cm high) were purchased from a Nursery. For the laboratory experiments, all plants were grown individually in UC soil mix [36] in plastic pots (50 mm × 50 mm × 120 mm). For the field experiment, all plants were grown individually in UC soil mix and cocopeat potting mix at a ratio of 1:1 in nursery bags (300 mm × 120 mm × 150 mm) in a glasshouse. Plants were placed in a field cage two weeks before the onset of field experiments to allow them to acclimatize to natural conditions.

### Parasitoid Handling

We used two- or three-day old mated female parasitoids. Newly formed parasitoid cocoons were collected and held individually in 100 ml plastic cups, each with a drop of honey and a water-soaked cotton dental wick. The newly emerged female parasitoids were caged with five males for 24 hours, with a drop of honey and water-soaked cotton dental wick, to ensure mating. The mated females were then isolated in glass vials (18 mm diam × 50 mm) with a drop of honey. Immediately before release, the individual parasitoids were primed by exposing them to feces collected from *E*. *postvittana* reared on an artificial diet in a Petri dish (80 mm diam). This stimulated the parasitoids with host-related cues that were not from any of the experimental plants. Individual parasitoids were then transferred to a clean vial (same as above) for release in the wind tunnel. The bottom half of the vial was filled with cotton to ensure the parasitoid did not move to the bottom and stay there.

### Choice Experiment

**Fig 2:**
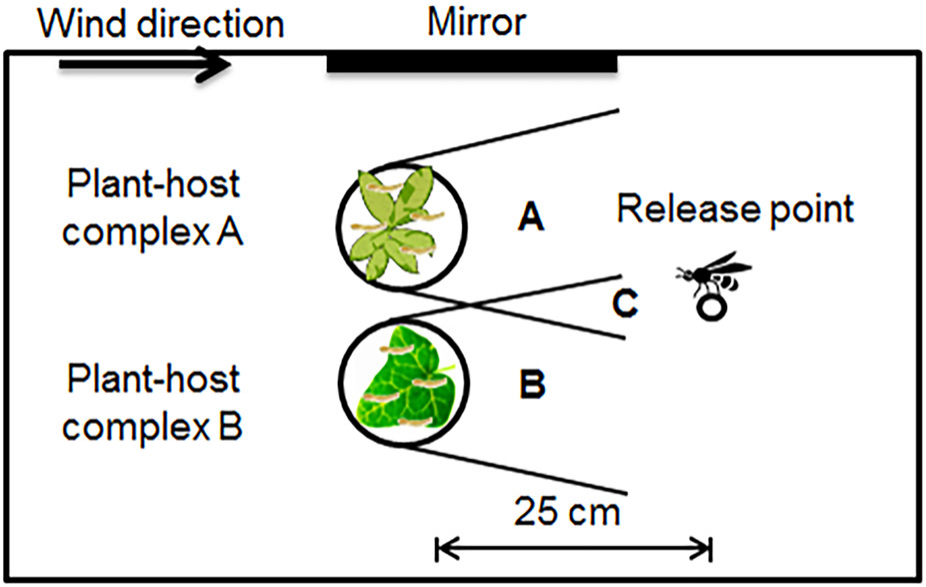
Experimental set-up of the wind tunnel for the choice test. There are three areas between the release point and the test plant: Area A has volatile cues from host plant one; Area B has volatile cues from host plant two; and Area C has volatile cues from both test plants.

An experiment was conducted to test the initial in-flight preference of the parasitoids for volatiles from different plants that were damaged by *E*. *postvittana*. There were four plant species, and therefore six pairs of volatile sources that were tested for each parasitoid species. Both parasitoid species were tested in dual-choice situations in which two volatile sources were placed in pairs in a wind tunnel at a wind speed of 20 cm/s at 21 ± 2°C (Fig 2; see [37] for wind tunnel details). To strengthen the volatile emissions and ensure continuous host-feeding damage on the plant leaves during the experiment, leaves of two plant species were infested with 20 second-instar *E*. *postvittana*. To reduce variations in morphology, texture, color, and structure and to ensure each parasitoid had a free and equal choice to fly to either of the target plants, the leaves of both plant species were placed in a metal screen tea ball (5.5 cm diam). Pilot tests indicated that only *D*. *tasmanica* would fly to host infested leaves that were enclosed in a tea ball. Therefore, to test the inflight preference of *T*. *unimaculatus*, plants infested with 20 second instar *E*. *postvittana* 24 h before the test were used. While different methods were used to test the innate preferences for host infested plants by these parasitoid species, this difference did not compromise our overall aim, which was to determine how host infested plants might differentially attract these parasitoids. Care was taken that all volatile sources from the same plant pair were used on the same day. To avoid plant-position bias effects, the positions of the two tea balls or the intact plants were randomized and with equal numbers of each test plant in each position. A single parasitoid was released from a glass vial on a stand 25 cm downwind from the tea balls or plants at approximately the same height, which were 25 cm above the floor of the wind tunnel and separated by 5 cm. The observation for each parasitoid lasted a maximum of five minutes. A ‘choice’ was recorded when a female landed on a tea ball or an intact plant. Wasps that did not respond within five minutes or landed elsewhere were recorded as ‘no response’. The experiments were conducted between 9:00 am and 4:00 pm, and each parasitoid was tested only once. Thirty-six parasitoids of each species were tested for each plant pair.

### Effects of Learning on Host-Plant Preference

An experiment was conducted with *D*. *tasmanica* to examine whether previous oviposition experience on host-infested plants alters the subsequent preference for host-induced plant volatiles. To provide female parasitoids with multiple oviposition experiences, they were allowed five sequential ovipositions on leaves of grape or *P*. *lanceolata*. A choice experiment was then conducted with an experimental design that was similar to the previous choice test. The parasitoids were observed in the wind tunnel to determine their landing preference between two plant species, of which one was the species of their previous oviposition experience. The experiment was conducted with 36 experienced parasitoids for each pair of plants and 36 naive parasitoids tested with the same pair of plants as a control, which also served to validate the results of the previous experiment. Due to insufficient numbers of *T*. *unimaculatus*, this parasitoid species was not included in this part of our study.

### Behavioral Assay

**Fig 3:**
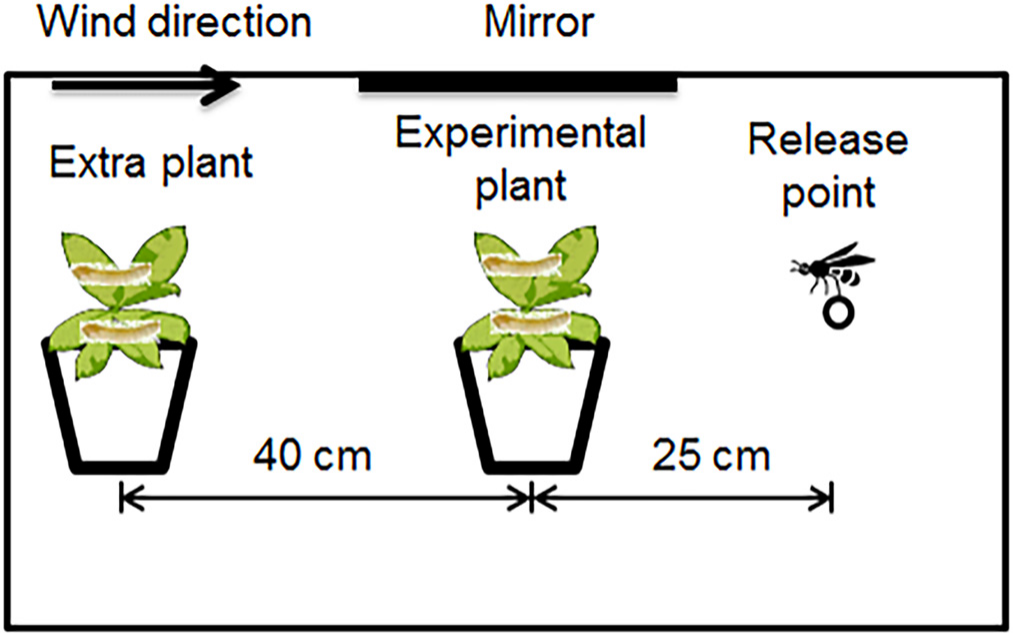
Experimental set-up of the wind tunnel for the behavioral assay. An individual plant infested with two second-instar larval *E*. *postvittana* was placed upwind of the parasitoid in the wind tunnel. To avoid the parasitoid spending excess time on the test plant, a second host plant of the same species and condition as the test plant was placed further upwind during each observation to provide an alternative landing place for the parasitoid.

To determine if parasitoid behavior varies among host plants, we also observed their foraging activities on the four plant species, which was influenced by the combined effects of all plant characteristics (plant chemistry and structure). This study was conducted in a wind tunnel (Fig 3). An individual plant infested with two second-instar *E*. *postvittana* was placed upwind of the parasitoid in the wind tunnel. To avoid the parasitoid spending excess time on the test plant, a second host plant of the same species and condition as the test plant was placed 40 cm upwind during each observation to provide an alternative landing place for the parasitoid. The second plant was also infested with two second-instar *E*. *postvittana*. Before the experiment, all of the leaves on the test plants were examined to check for the position and number of host larvae, host damage, and frass on the plants. A single parasitoid was released from a glass vial 25 cm downwind from the host infested plant. The foraging behavior of individual parasitoids on the downwind host-infested plant was then observed, with the observations lasting until the parasitoid left the plant to either another location in the wind tunnel or the alternative plant. For both parasitoid species, twenty wasps were observed for each host-plant species, and each parasitoid was observed only once. The order of testing the plant species was randomized.

Based on preliminary observations, a catalogue of female behavior for *D*. *tasmanica* and *T*. *unimaculatus* searching was constructed (Table 1). The mean duration for each type of behavior was calculated from when a parasitoid first landed on the host plant. According to a pilot experiment, a common host defensive behavior to avoid parasitoid attack is to drop from the plant. Therefore, the dropping behavior of the host larvae was also recorded, as were the number of larvae that were stung. Each day, observations were conducted between 10:00 am and 4:00 pm. Parasitoid behavior was recorded with the Observer XT ver. 11 software package (Noldus Information Technology B.V., Wageningen, the Netherlands). The egg hatching time of *D*. *tasmanica* and *T*. *unimaculatus* are about two and four days after egg laying, respectively [38]. Therefore, the larvae stung by the *D*. *tasmanica* and *T*. *unimaculatus* were dissected two and four days after the experiment, respectively, to determine the parasitism rate.

**Table 1:** A catalogue of behavior of *Dolichogenidea tasmanica* and *Therophilus unimaculatus*.

Event	Description
**Antennating**	Walking with antennae touching and sweeping along the substrate
**Flying**	Any airborne activity
**Grooming**	Preening any part of the body
**Probing**	Walking while drumming the substrate with antennae and jabbing with the ovipositor
**Stationary**	Standing still with moving antennae
**Stinging**	Stinging a host with the ovipositor
**Walking**	Walking with antennae not touching the substrate
* **Pulling**	Pulling the thread of a hanging host larva and hoisting it up

* *T*. *unimaculatus* only

### Field Experiment—Parasitism of *E*. *postvittana* on Four Plant Species

**Fig 4:**
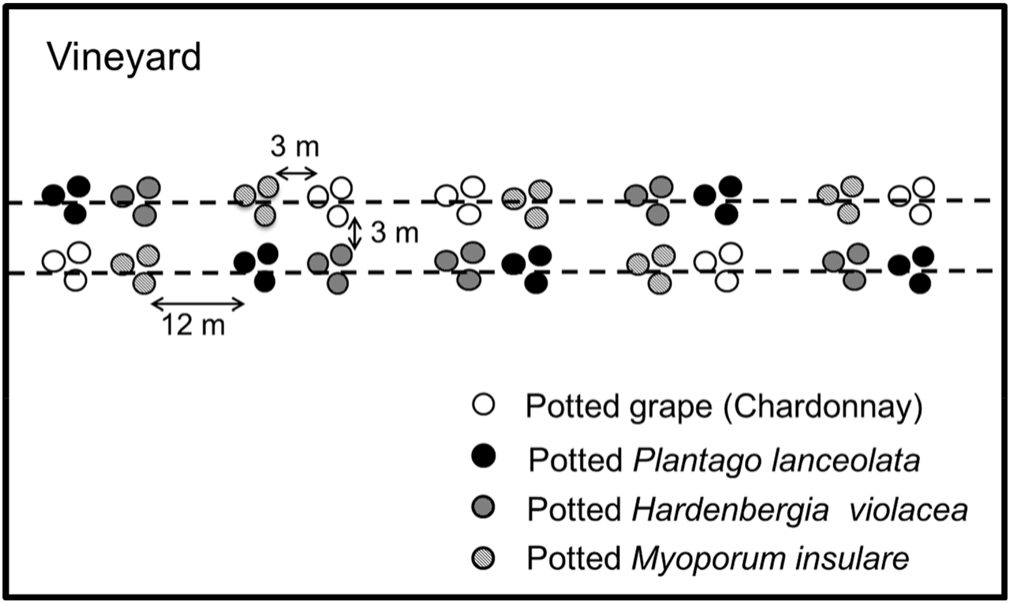
Scheme of the experimental set-up in vineyards. Three pots of each plant species were arranged together and three meters away from the other plants within the sampling point. The sampling points were 12 m apart. Dashes lines indicate the vine rows.

To evaluate whether the four plant species influence the levels of parasitism by *D*. *tasmanica* and *T*. *unimaculatus* in vineyards, a field experiment was conducted at two sites (vineyard A: 35°13' S, 138°39' E; vineyard B: 35°16' S, 138°37' E) and repeated twice at each vineyard between February and March 2013. An orthogonal split-plot design was used where the vineyards were considered random blocks, the plant species were the main-plot factor, and the repeated visits were the split-plot factor. In each vineyard, the four different species of potted plants infested with first-instar *E*. *postvittana* (around 20 larval *E*. *postvittana* on each plant) were placed in five sets of quadruplets (sample unit = 3 plants/species) inside the vineyard and 20 m from the border (Fig 4). At each site, 60 plants were placed in the field and left for two weeks of free access to wild parasitoids. The plants were placed in the two vineyards on consecutive days. After two weeks, the plants were removed and replaced with a fresh pair of plants. The larvae on each plant were collected and reared in 440 ml plastic containers at 22 ± 2°C under a 14 L:10 D light/dark cycle in an insect-rearing room. The parasitism rate and fate of the larvae (dead, parasitized, or pupated) were recorded.

### Statistical Analysis

To determine which host plant was preferred by the parasitoids, the choice within an experiment was analyzed using a binomial test, with 0.5 as the null hypothesis. To examine if learning can alter the landing preference of the parasitoids, chi-squared tests with Cochran's correction for continuity [39] were used to compare the landing choices of the naive/control and experienced wasps.

The mean proportion of time devoted to each type of behavior after landing on a plant was calculated for each parasitoid species on each plant species. The proportion of time that wasps were engaged in each behavior was calculated for each individual, and differences among the plant species for each parasitoid were analyzed with Kruskal-Wallis tests (IBM SPSS Statistics v. 20, IBM-SPSS Inc., Chicago, IL).

In addition, Chi-square tests were also used to determine if differences in parasitism rates and host defensive behaviors (larvae dropping) among four plant species were statistically significant. If the null hypothesis was not rejected, a retrospective power analysis for Chi-square was carried out (Functions CHISQ_POWER and CHISQ_SIZE, default power = 0.8, www.real-statistics.com) to determine the required sample size with the significance level of 0.05.

To analyse the factors affecting parasitism in the experimentally introduced *E*. *postvittana*, the data from field experiments were modelled with orthogonal split-plot general linear models with the GLM procedure in the statistical package GenStat for windows 15^th^ Edition (VSN International, Hemel Hempstead, UK.). For all experiments, the fractions of larvae that were parasitised by all parasitoids on each plant species in each quadruplet were calculated from the pooled numbers; these numbers were treated as the dependent variables. The arcsine transformation [39] was used to analyse the parasitism data. The Bonferroni adjustment was used for post-hoc multiple comparisons of means. The level of significance was set at 0.05.

## Results

### Choice Experiment

**Fig 5:**
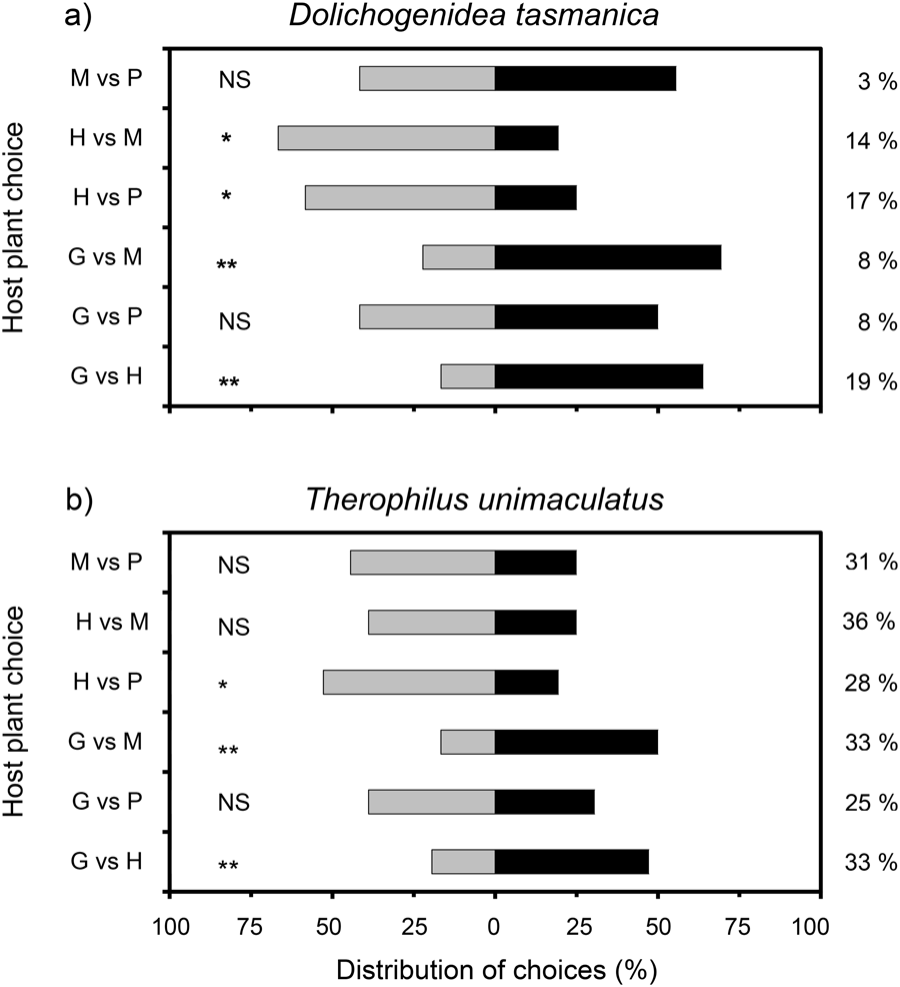
Distribution of choices made by *Dolichogenidea tasmanica* (a) and *Therophilus unimaculatus* (b) in response to plants infested with second instar *Epiphyas postvittana*. Within each choice test, the per cent of parasitoids that made no choice is shown at the right. Host plants: P, *P*. *lanceolata*; G, grape; H, *H*. *violacea*; M, *M*. *insulare*. Asterisks indicate a significant difference between the targets (binomial test; *P < 0.05, **P < 0.005). *NS*, not significant.

Parasitoids had varied preferences for landing in the dual-choice experiments. Female *D*. *tasmanica* preferred to land on one species combination over another in four of the six plant-host-complex pairings (Fig 5a). *H*. *violacea* was preferred over *M*. *insulare*, grape and *P*. *lanceolata*, and *M*. *insulare* was preferred over grape. *T*. *unimaculatus* females showed preference in three of the six pairings (Fig 5b). *H*. *violacea was preferred* over grape and *P*. *lanceolata*, and *M*. *insulare* was preferred over grape.

### Effects of Learning on Host Plant Preference

**Fig 6:**
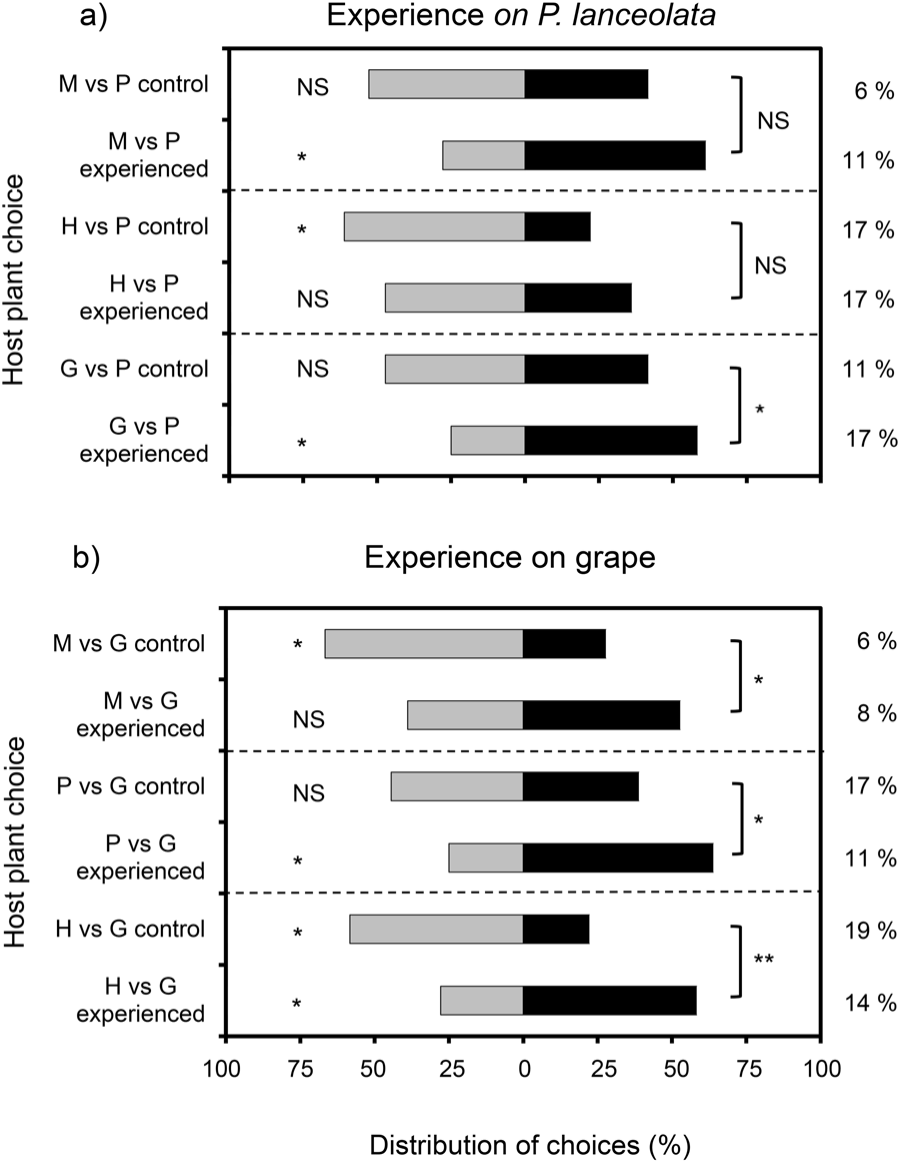
Distribution of choices made by *Dolichogenidea tasmanica* in response to different species of plants infested with *Epiphyas postvittana*. The female *D*. *tasmanica* had previous oviposition experience on *Plantago lanceolata* (a) or grape (b). Host plants: P, *P*. *lanceolata*; G, Grape; H, *H*. *violacea*; M, *M*. *insulare*. Control groups were naive parasitoids. Within each choice test, the per cent of parasitoids that made no choice is shown at the right. Asterisks on the left side of the table indicate a significant difference between the targets (binomial test; *P < 0.05, **P < 0.005). Asterisks on the right side of the table indicate a significant difference between the experimental and control groups (χ^2^ tests with Cochran’s correction for continuity; *P < 0.05). NS, not significant.

Oviposition experience on a plant affected subsequent plant preferences in *D*. *tasmanica*. In the experiments that involved experience on *P*. *lanceolata*, the change in preference between control and experienced wasps was statistically significant only when the comparison between them involved the choice between *P*. *lanceolata* and grape (Fig 6a). However, there was a statistical change in the degree of preference between species in all paired groups of control and experienced wasps. For example, naive wasps displayed no preference between *M*. *insulare* and *P*. *lanceolata*, but wasps that had experience on *P*. *lanceolata* significantly preferred to land on it. Likewise, naive wasps that were presented with choice between *H*. *violacea* and *P*. *lanceolata* preferred to land on *H*. *violacea*, but this preference was not displayed by wasps that had experience on *P*. *lanceolata*. Oviposition experience on grapes led to more frequent landing on grapes by *D*. *tasmanica* in the presence of each of the other three plant species (Fig 6b). Interestingly, the preference of naive *D*. *tasmanica* for *H*. *violacea* over grape was reversed after females had oviposition experiences on hosts that feeding on grapes.

### Behavioral assay

**Fig 7:**
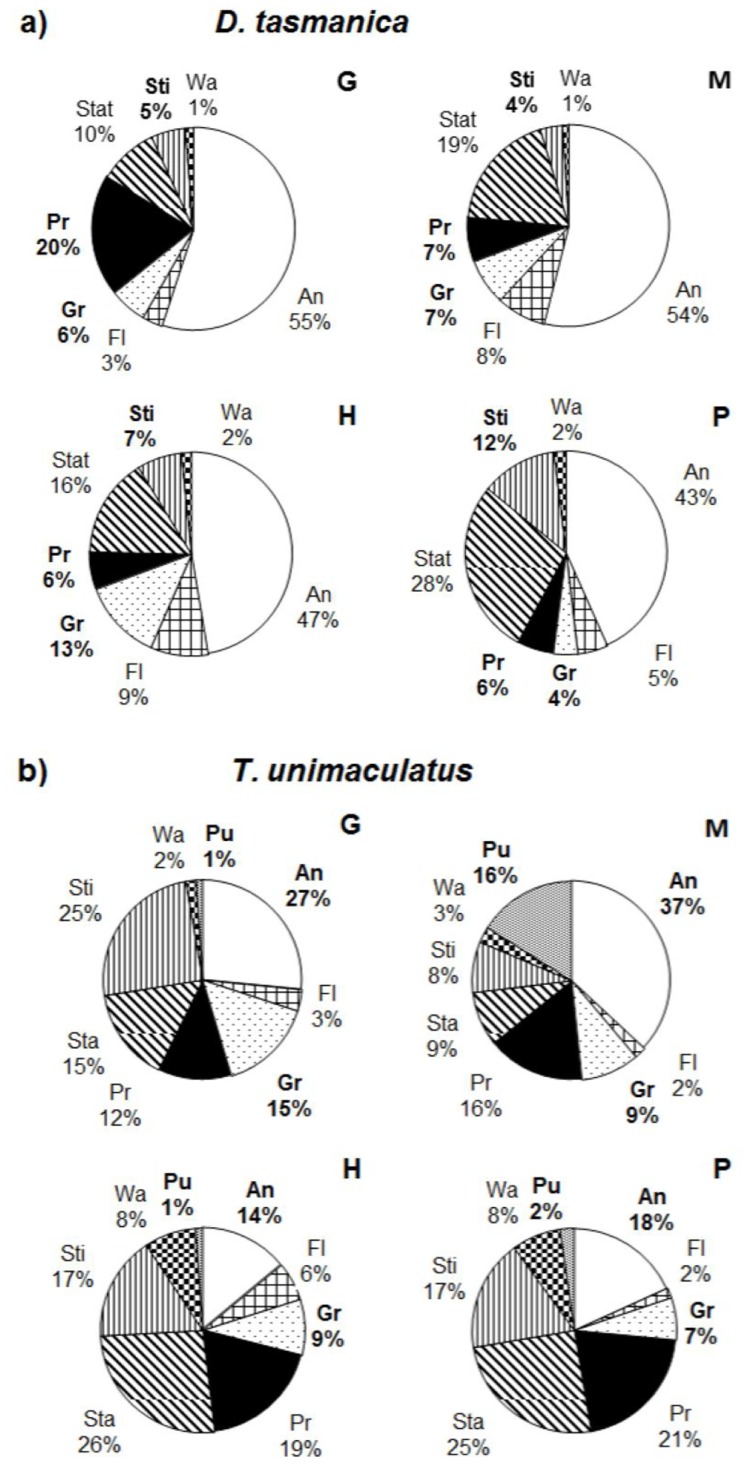
Proportion of time spent by *Dolichogenidea tasmanica* (a) and *Therophilus unimaculatus* (b) while foraging on different plant species. Behaviors (see Table 1 for definitions): An, antennating; Fl, flying; Gr, grooming; Pr, probing; Pu, pulling; Sta, stationary; Sti, stinging; Wa, walking. Host plants: P, *P*. *lanceolata*; G, Grape; H, *H*. *violacea*; M, *M*. *insulare*. Bold behavior characters indicate significant differences among plant species (Kruskal Wallis tests; P < 0.05).

**Fig 8:**
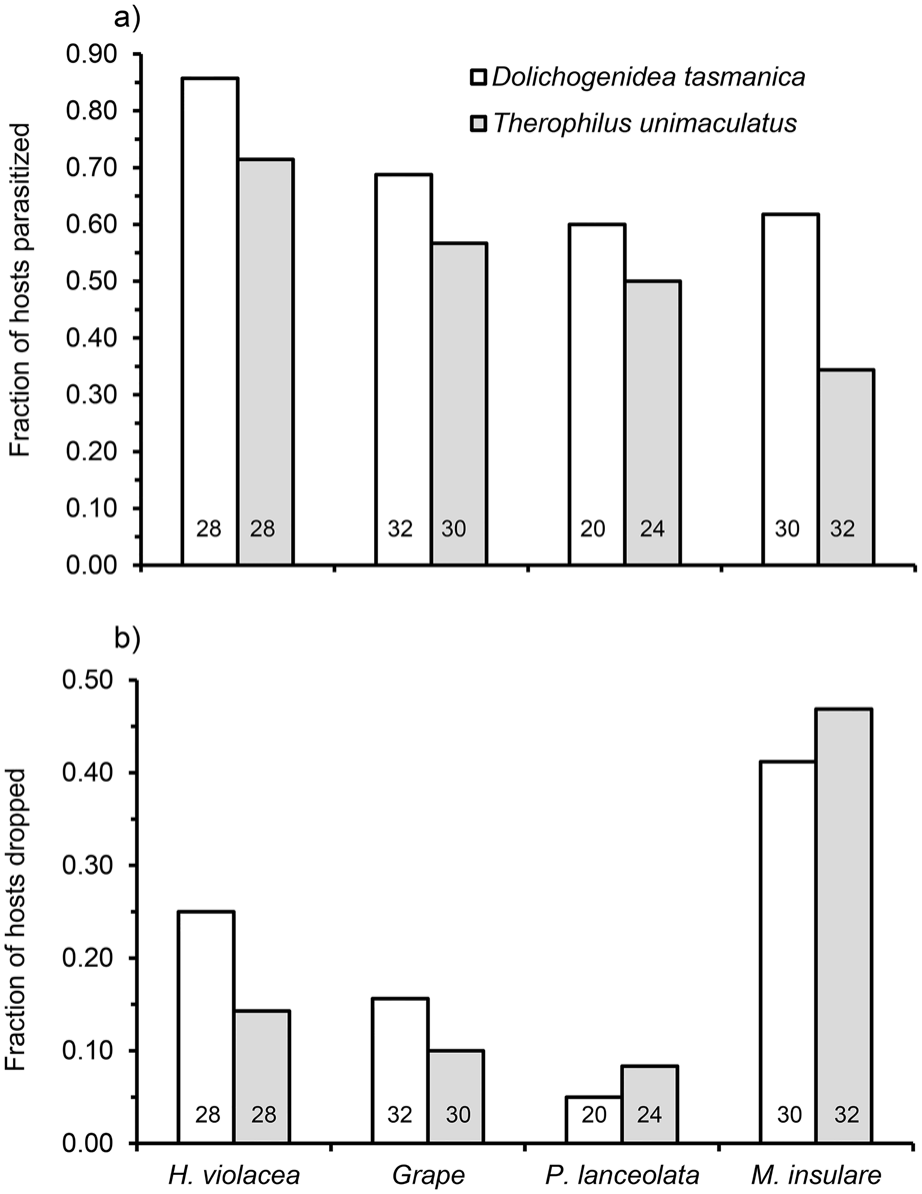
Fraction of second instar *Epiphyas postvittana* that a) dropped from plants when approached by parasitoids and b) were parasitized by *Dolichogenidea tasmanica* and *Therophilus unimaculatus* among four host-plant species in the behavioral assay. The number of larvae that dropped/total differed significantly among four host plant species (*D*. *tasmanica*, χ^2^ = 10.80, P = 0.013; *T*. *unimaculatus*, χ^2^ = 18.15, P = 0.0004). Number of larvae parasitised was not different among host plant species for *D*. *tasmanica* (χ^2^ = 5.27, P = 0.153). Chi-square power analysis (default power = 0.8) indicated that the sample size must be increased by 2.06 times before the experimental results would be likely to produce statistically significant differences. No. of larvae parasitised was higher on *H*. *violacea* than the other three host plant species for *T*. *unimaculatus* (χ^2^ = 8.51, P = 0.037). Numbers within each bar indicate the sample sizes.

**Fig 9:**
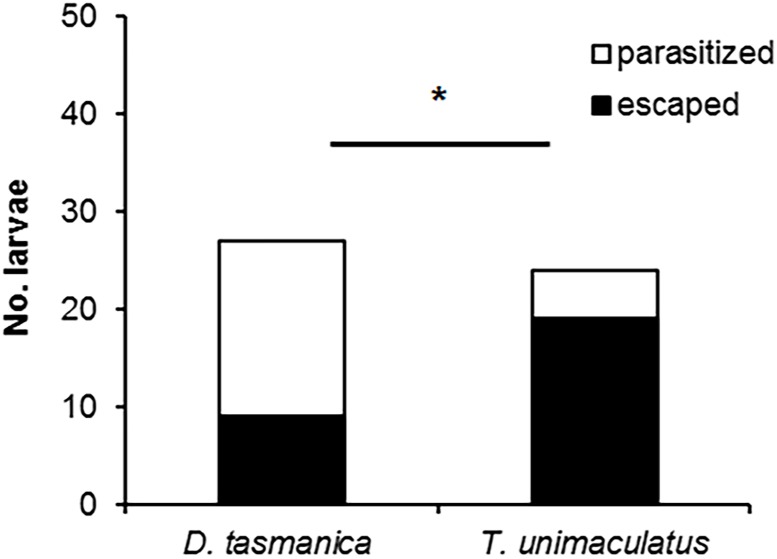
Number of dropped larvae parasitized by *Dolichogenidea tasmanica* and *Therophilus unimaculatus* in behavioral assay. χ^2^ test; *P < 0.05.

The behavioral profiles of both parasitoids varied among the four host-plant species (Fig 7; raw data in S1 Table). The fractions of time *D*. *tasmanica* engaged in stinging, probing and grooming differed among plant species, while the fractions of time *T*. *unimaculatus* allocated to pulling, antennating and grooming differed among species. The parasitism rate of *T*. *unimaculatus* on *H*. *violacea* was higher than on the other three plant species. The parasitism rate by *D*. *tasmanica* on *H*. *violacea* was higher than on other plant species but this difference was not statistical significant, possibly due to low statistical power (Fig 8a). The most common way that larval *E*. *postvittana* avoided attack was to drop from the plant on a silk thread when contacted by a parasitoid. From our laboratory observations, more larvae dropped from *M*. *insulare* than from the other plants (Fig 8b). Among the dropping hosts pooled among all plants, significantly more were parasitized by *D*. *tasmanica* than by *T*. *unimaculatus* (Fig 9). The two parasitoid species responded differently toward the escaping hosts. When encountering an escaping host hanging from a silk strand, *D*. *tasmanica* immediately followed the larva or attacked it on the thread, or searched for few seconds, flew to locate it, and then attacked the larvae on the thread. If the host dropped to the ground, some of the *D*. *tasmanica* flew or walked to the ground and searched for the host. In contrast, *T*. *unimaculatus* displayed hauling behavior when encountering a dropping larva, continuously reeling the silk thread with its legs up to pull the hanging larva back to the plant, even though in many cases the larva would drop to the ground. No *T*. *unimaculatus* was observed following the dropping host or searching for the larvae on the ground. *D*. *tasmanica* found and attacked the dropping hosts more successfully than did *T*. *unimaculatus* (Fig 9).

### Field Experiment—Parasitism of *E*. *postvittana* on Four Plant Species

**Fig 10:**
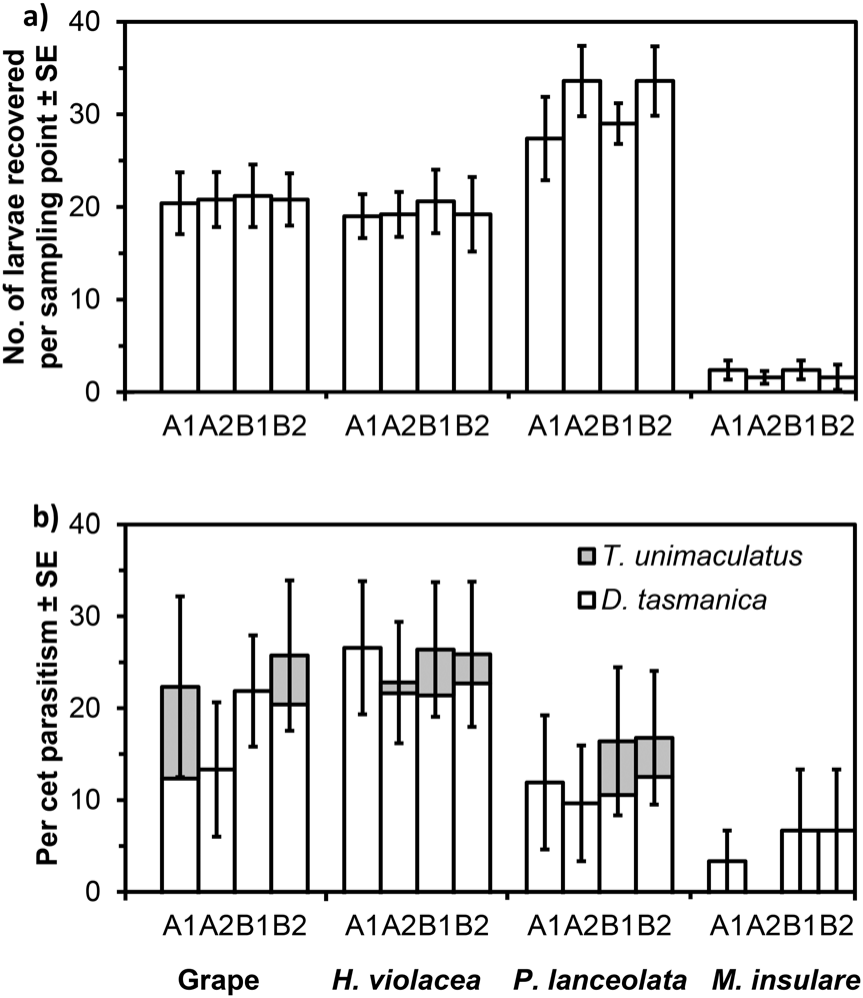
Mean number of larval *Epiphyas postvittana* recovered (a) and parasitism of *E*. *postvittana* (b) in the field experiment. The larvae were feeding on grape, *H*. *violacea*, *P*. *lanceolata*, and *M*. *insulare*. Data are expressed as overall means ± standard error.

The number of larval *E*. *postvittana* that were found on each three-pot group of plant species differed significantly among the four plant species (Fig 10a; F_3,76_ = 70.06, P < 0.05; raw data in S2 Table). A low number of larval *E*. *postvittana* was recovered from *M*. *insulare* compared with other plant species in both replications.

Only two parasitoid species, *D*. *tasmanica* and *T*. *unimaculatus*, were recovered from the larval *E*. *postvittana* on plants placed in the vineyards. No *T*. *unimaculatus* were collected from larvae on *M*. *insulare*. The angular-transformed parasitism rates for *D*. *tasmanica* and overall parasitism differed among plant species (Fig 10b; *D*. *tasmanica*, F_3, 76_ = 12.41, P < 0.05; overall parasitism, F_3, 76_ = 34.64, P < 0.05). However, the angular-transformed parasitism rates for *T*. *unimaculatus* were not different among plant species (Fig 10b; *T*. *unimaculatus*, F_3, 76_ = 2.52, P = 0.234), which reflects the low and inconsistent appearance of this species between sites and dates. Overall parasitism on *M*. *insulare* was significantly lower than on grape and *H*. *violacea*, but there were no detectable differences in parasitism by *D*. *tasmanica* among plant species when Bonferroni-test adjustments were made to multiple comparisons.

## Discussion

The results of this study indicate that herbivore infested plants affect the foraging behavior and efficiency of both *D*. *tasmanica* and *T*. *unimaculatus*. Although we did not directly measure and compare the herbivore-induced plant volatile profiles of the plant species we tested, the effect of host induced plant volatiles were directly observed through comparing the responses of the parasitoids to paired host-infested plants. The results indicate both parasitoids have innate preferences for plant species. The Australian native plant, *H*. *violacea*, was the most attractive species to both parasitoids. Studies have demonstrated that among a range of plant species infested with the same herbivore, the host-induced volatiles differ in both specificity and quantity, and affect the natural enemies differently [9,40,41]. The native plant *H*. *violacea* was more attractive to the parasitoids, but the exotic plants *P*. *lanceolata* and grape, which have established recent interactions with the second and third trophic levels, also attracted the parasitoids. This suggests adaptability by these generalist parasitoids as well as innate responses to common characteristics of plants. In this study, the plant species on which parasitoids were reared could have potentially affected their behavior [42]. However, our main aim was to evaluate how plant species could generally affect the behavior of parasitoids that are foraging in a diverse landscape. We would not expect any potential bias from rearing on a selected plant species to affect this broader evaluation.

Previous oviposition experience with hosts that were feeding on grape and *P*. *lanceolata* both affected the subsequent plant preference of *D*. *tasmanica* in the dual-choice tests (Fig 6a–6b). When all statistical analyses are considered together, the results indicate that *D*. *tasmanica* is more likely to fly to a particular plant species after it has experience with parasitizing a host on it. The magnitude of such shifts in preference is likely to depend on the degree of innate preference, the level of experience and the characteristics of the plants involved. From these results, we hypothesize that *T*. *unimaculatus* can also learn to associate hosts with some plants as a result of experience. Assuming this is the case, the experiences of both species could reinforce their preferences with hosts on innately preferred and common host plant species in or near vineyards. On the other hand, experience on previous less preferred plants may lead to a reduction in preference for a host plant that is preferred by a naive parasitoid.

*D*. *tasmanica* and *T*. *unimaculatus* allocated their times engaged in various types of behavior differently when foraging on different plant species. In addition, *D*. *tasmanica* spends relatively more time antennating and probing and less time stinging than *T*. *unimaculatus* (Fig 7). These results indicate that a combination of plant characteristics influences the foraging behavior of these parasitoids. Many studies have demonstrated that plant structure can affect parasitoid foraging efficiency [43]. The size, heterogeneity and connectivity of plants have been shown to affect parasitoid foraging success, as confirmed both by modelling the impact of plant structure on parasitism rates based on artificial plants and by experiments with real plants [44]. The probability of parasitoids and predators encountering a host or prey generally decreases with an increase in plant structural complexity [11,14,16,20,45,46], plant size [47], and plant surface area or volume [48–50].

Our results also indicate that the plant species affect the defensive behavior of the host (Fig 8), and therefore indirectly affect the foraging efficiency of parasitoids (Fig 9). Host larvae falling from plants may be at a higher risk of encountering other predators or parasitoids [51]. Because of this, plant species that facilitate escaping behavior in the field may not support large numbers of parasitoids.

*D*. *tasmanica* was found to be the dominant species that parasitizes larval *E*. *postvittana* in vineyards. Both *D*. *tasmanica* and *T*. *unimaculatus* were found in the field experiment, but *D*. *tasmanica* parasitised the majority of the sentinel larval *E*. *postvittana* in the vineyards. Parasitism by *D*. *tasmanica* was consistent between sites and dates, while parasitism by *T*. *unimaculatus* was inconsistent and low (Fig 10b). This is in line with a previous study that indicated the dominance of *D*. *tasmanica* in vineyards [33]. Under field conditions, host-related chemical cues are the main long-distance attractor for parasitoids, which could influence their foraging efficiency. Plants could be a key factor that affects the activity of parasitoids in certain habitats. Studies have suggested that plants which are attractive to parasitoids are associated with a higher parasitism rate under field conditions [10]. There is independent evidence that parasitism by *D*. *tasmanica* varies significantly among plant species [52], which is consistent with the results of our field experiment. Moreover, our behavioral assays indicated that plants vary in their level of attraction to females, and this can be influenced by a wasp's experience. In one behavioral assay, parasitism rates did not differ among plant species, but this non-significant result is likely to be due to low statistical power (Fig 8a). This indicates that differences among plants may not be pronounced when some species combinations are compared.

Research has indicated that successful foraging on certain host plants can narrow a parasitoid’s foraging range through learning [53]. The field experiment was carried out in vineyards, where the main background plants were grapes and *P*. *lanceolata* was a common ground cover plant. Although *H*. *violacea* could be the preferred host plant species of the naive wasp, the parasitoids may gain experiences more frequently after attacked hosts feeding on the more abundant and naturally occurring grape or *P*. *lanceolata* in vineyards and thus strengthen their habitat preference. This could happen for both *D*. *tasmanica* and *T*. *unimaculatus* that were attracted to vineyards. However, when both parasitoids are active in a vineyard, their competitive interactions would influence their foraging success and abundance [38]. In this case, the more abundant parasitoid species should be found to have a competitive advantage over its competitors.

Results from our field experiment showed the number of larval *E*. *postvittana* recovered from the four different plant species varies. It is noticeable that much smaller numbers of larval hosts were recovered from *M*. *insulare* compared to the other three plant species (Fig 10a). Studies have indicated that plants infested with a relatively high density of host larvae should attract more parasitoids [54]. Therefore, the smaller number of host larvae could have negatively affected the parasitism of *E*. *postvittana* on *M*. *insulare*. It is also possible that larvae could not defend themselves as effectively from parasitoids and predators on *M*. *insulare*. In the case of parasitoid attack, larvae that dropped from the plant may have failed to return to it more frequently than on other host plant species.

In conclusion, plants influence host availability and attract the two parasitoids differently. Different plant species provide different levels of protection for larval *E*. *postvittana* and thereby affect the foraging behavior and efficiency of parasitoids that attack it. Results of our field experiment indicate *D*. *tasmanica* is the dominant parasitoid in vineyards, and plant species affect parasitism by this parasitoid species. Putting these effects together, we conclude plants are likely to affect the habitat preferences and distributions of these parasitoids that share the same hosts. In this study we investigated tri-trophic interactions involving two Australian native plants, *H*. *violacea* and *M*. *insulare*, as well as the introduced exotics, *P*. *lanceolata* and grape. This approach revealed how plants generally affect the parasitoid-host interactions in real vineyard ecosystems, in which numerous plant species are involved in the interactions between the herbivore and its associated parasitoid species.

Future research should address the question of whether adding specific plant species that are preferred by parasitoids could increase the suppression of *E*. *postvittana* in vineyards. To strengthen the activity of parasitoids that are already active in vineyards or attract more parasitoids into vineyards, it is necessary to evaluate a wider range of supplementary plant species, especially native and perennial species. Understanding the interactions at the tri-trophic level of plant, pest and natural enemies should help us “ecologically engineer” vineyards [55] to promote the activity of these parasitoids and therefore enhance the ecosystem service of biological control [56].

## Supporting Information

S1 Table Behaviour assay data. (CSV)

S2 Table Field experiment data. (CSV)
